# Thioredoxin-related transmembrane protein 2 (TMX2) regulates the Ran protein gradient and importin-β-dependent nuclear cargo transport

**DOI:** 10.1038/s41598-019-51773-x

**Published:** 2019-10-25

**Authors:** Ami Oguro, Susumu Imaoka

**Affiliations:** 1 Department of Biomedical Chemistry, School of Science and Technology, Kwansei Gakuin University, Sanda, Japan; 2 Program of Biomedical Science, Graduate School of Integrated Sciences for Life, Hiroshima University, Hiroshima, Japan

**Keywords:** Transport receptors, Chaperones

## Abstract

TMX2 is a thioredoxin family protein, but its functions have not been clarified. To elucidate the function of TMX2, we explored TMX2-interacting proteins by LC-MS. As a result, importin-β, Ran GTPase (Ran), RanGAP, and RanBP2 were identified. Importin-β is an adaptor protein which imports cargoes from cytosol to the nucleus, and is exported into the cytosol by interaction with RanGTP. At the cytoplasmic nuclear pore, RanGAP and RanBP2 facilitate hydrolysis of RanGTP to RanGDP and the disassembly of the Ran-importin-β complex, which allows the recycling of importin-β and reentry of Ran into the nucleus. Despite its interaction of TMX2 with importin-β, we showed that TMX2 is not a transport cargo. We found that TMX2 localizes in the outer nuclear membrane with its N-terminus and C-terminus facing the cytoplasm, where it co-localizes with importin-β and Ran. Ran is predominantly distributed in the nucleus, but TMX2 knockdown disrupted the nucleocytoplasmic Ran gradient, and the cysteine 112 residue of Ran was important in its regulation by TMX2. In addition, knockdown of TMX2 suppressed importin-β-mediated transport of protein. These results suggest that TMX2 works as a regulator of protein nuclear transport, and that TMX2 facilitates the nucleocytoplasmic Ran cycle by interaction with nuclear pore proteins.

## Introduction

Thioredoxin-related transmembrane proteins (TMXs) are protein disulfide isomerase (PDI) family members and possess a transmembrane region. TMX1 was first identified in 2001 by Matsuo *et al*.^1^. TMX proteins are integral transmembrane proteins that localize predominantly in the ER, but are also found in mitochondria-associated membranes.^2^ TMX1, TMX3, and TMX4 have a conserved active site, Cys-X-X-Cys (CXXC), for oxidoreductase activity in a thioredoxin-like domain^3–5^, and regulate protein quality in the ER^6,7^, ER-mitochondria Ca^2+^ flux, platelet function^8^, eye development^9^, and so on. On the other hand, TMX2 has a single cysteine in the conserved SXXC motif and lacks oxidoreductase activity. TMX2 cDNA was first isolated in 2003, and TMX2 mRNA has been found in a variety of human tissues, with the highest levels in the heart, brain, liver, kidney, and pancreas^10^. TMX2 protein was found in the supernatant of cultured bovine periosteal cells^11^. A recent TMX2 knockout experiment in primary mouse cortical neurons showed the protective function of TMX2 from ER stress^12^. Apart from these findings, however, there is no information about TMX2, and its target proteins and functions remain unknown. Though PDI family proteins, ERp44 and ERp29, also lack the CXXC motif or oxidoreductase activity, these proteins are known to serve as a scaffold or coat protein for protein transport; ERp44 has a role in the transport of immature proteins from the Golgi apparatus to the ER for the quality control of secretory proteins^13^, and ERp29 regulates ER membrane penetration of the polyomavirus coat protein to access the cytosol^14^.

In this study, we searched for TMX2-binding proteins, and identified several nuclear transport proteins including importin-β and the Ras-related small nuclear GTPase, Ran. Nucleocytoplasmic trafficking through the nuclear pore complex is a fundamental cellular process in eukaryotic cells. Importin-β family proteins serve as adaptors of cargo proteins that have a nuclear localization signal (NLS) with importin-α, or directly bind to cargo proteins and transport them through the nuclear pore complex into the nucleus^15,16^. In the nucleus, the binding of importin-β and the GTP-bound form of Ran (RanGTP) releases the cargo. RanGTP is also essential for the nuclear export of cargo proteins with Chromosomal Maintenance 1 (CRM1), which is also known as Exportin-1, and for recycling importin-β from the nucleus to the cytosol^17^. Hence, levels of both the total Ran protein and GTP-bound Ran are abundant in the nucleus while small amounts are present in the cytoplasm, and this nucleus/cytosol Ran gradient helps properly drive the cargo transport cycle. It has been shown that impairment of the transport cycle accompanying disruption of the Ran gradient induces cell senescence^18,19^, and may be related with diseases such as Hutchinson-Gilford Progeria Syndrome^20,21^.

In the present study, we found new functions of TMX2 in the maintenance of the Ran protein gradient and regulation of importin-β-dependent cargo transport.

## Results

### TMX2 interacts with importin-β and Ran but is not a transport cargo of importin-β

**Figure 1:**
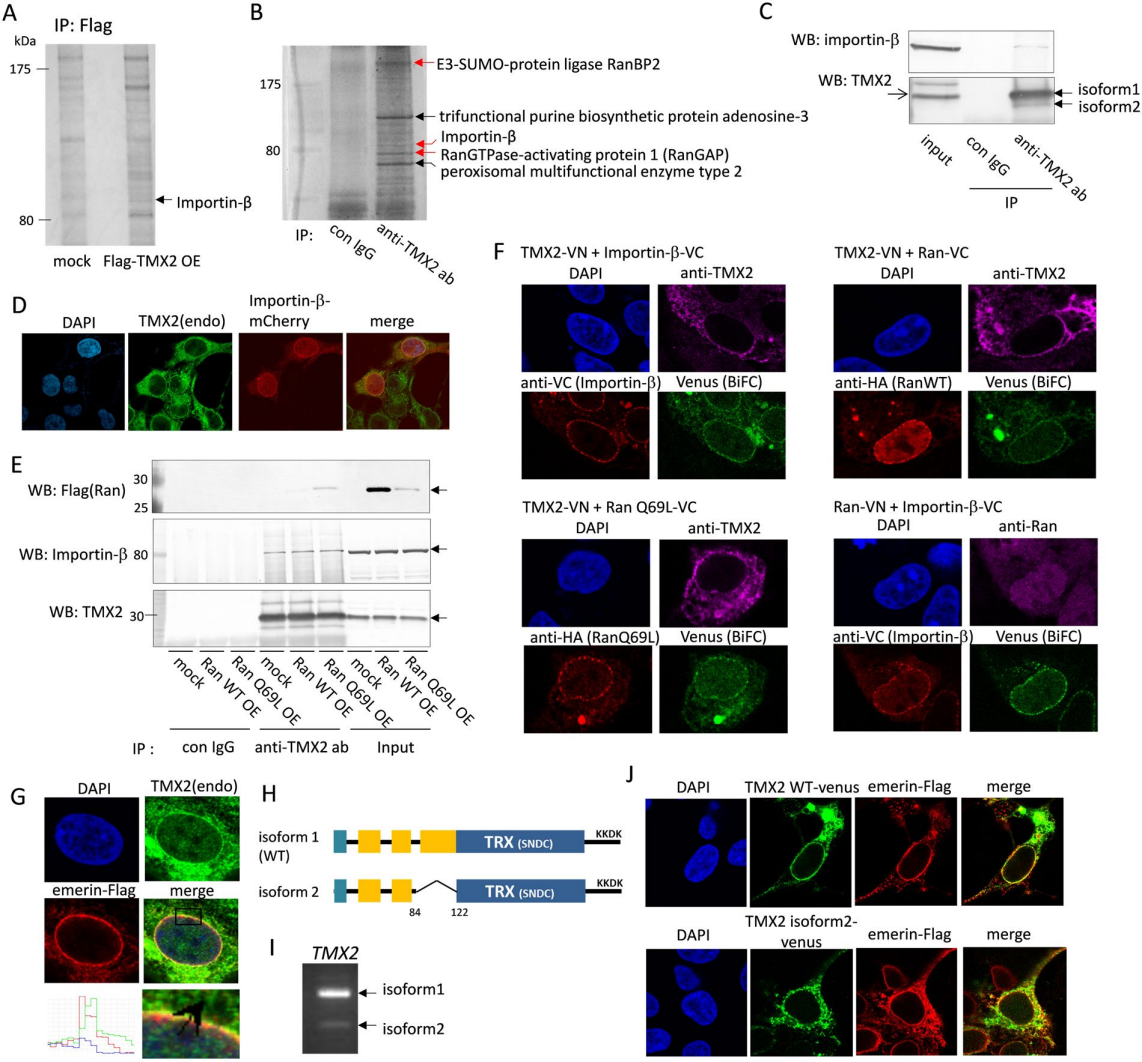
TMX2 interacts with nuclear import protein. (**A**) Flag-TMX2 was overexpressed in HEK293 cells and immunoprecipitated with anti-Flag antibody. The band indicated by the arrow was analyzed by LC-MS. (**B**) Endogenous TMX2-intereacting proteins in HEK293 cells were immunoprecipitated with anti-TMX2 antibody and analyzed by LC-MS. (**C**) HEK293 cell lysates were immunoprecipitated with anti-TMX2 antibody and analyzed by western blot with anti-importin-β antibody. (**D**) mCherry-importin-β was expressed in HEK293 cells and immunostained with anti-TMX2 antibody. (**E**) Flag-Ran WT or -Ran Q69L mutant was overexpressed in cells and immunoprecipitated with anti-TMX2 antibody. (**F**) TMX2-VN was co-expressed with importin-β-VC, HA-RanWT-VC, or HA-RanQ69L-VC in HEK293 cells, and the overexpression of these proteins were confirmed by anti-TMX2, anti-VC, or anti-HA antibody. Venus fluorescence as BiFC signal indicates the interaction of these fusion proteins. BiFC signal was also detected by co-expression of Ran-VN and importin-β-VC as a positive control. (**G**) Co-localization of endogenous TMX2 and Flag-emerin was analyzed by immunofluorescence. (**H**) A diagram of TMX2 WT and isoform 2, which lacks part of the transmembrane region. (**I**) TMX2 WT and isoform 2 cDNA were amplified by PCR from HEK293 cDNA with the primer sets at 191–507th of TMX2 WT nucleotide. (**J**) Venus fused with TMX2 WT or isoform 2 was expressed in HEK293 cells, and co-localization with emerin-Flag was analyzed.

TMX2 isoform 1 cDNA (NM_015959.4), which encodes 296 amino acids, was isolated from Hep3B cells. To identify the TMX2-interacting protein, Flag-TMX2 was overexpressed in HEK293 cells, and immunoprecipitated with anti-Flag antibody. The cell lysates were analyzed by SDS-PAGE and silver staining. Protein bands exhibiting differential immunoprecipitation patterns between mock and Flag-TMX2 fractions were analyzed by mass spectrometry, and the protein band indicated by the arrow was identified as Karyopherin Subunit Beta 1 (KPNB1/importin-β) (Fig. 1A). Importin-β is a nuclear transport receptor that imports cargoes from the cytosol into the nucleus. Immunoprecipitation was also done with anti-TMX2 antibody using HEK293 cell lysates to search for proteins interacting with endogenous TMX2. Several proteins including importin-β were identified; cytoplasmic nuclear pore complex proteins, namely the SUMO E3 ligase complex RamBP2 and the Ran GTPase-activating protein (RanGAP), which regulate hydrolysis of RanGTP to RanGDP, were also identified (Fig. 1B). From these results, TMX2 was anticipated to have a role in nuclear transport. The interaction between endogenous TMX2 and importin-β was confirmed by immunoprecipitation with anti-TMX2 antibody and immunoblotting with anti-importin-β antibody (Fig. 1C). Endogenous TMX2 mainly localized in the ER, but also co-localized with mCherry-fused importin-β in the peripheral nuclear envelope (Fig. 1D). Next, we investigated whether TMX2 is a cargo protein of importin-β. Cargo proteins transported into the nucleus by importin-α/β are released in the nucleus by binding of importin-β with the GTP-bound form of Ran, RanGTP. Overexpression of Ran WT or RanQ69L, which is a mutant locked in the GTP-bound state, did not decrease the binding between TMX2 and importin-β (Fig. 1E). In addition, overexpression of importin-β did not affect localization of TMX2 (Fig. 1D), and TMX2 did not accumulate in the nucleus in the presence of an inhibitor of exportin CRM1, leptomycin B (see Supplementary Fig. S1). These results indicate that while TMX2 does interact with importin-β, TMX2 is not a cargo of importin-β to be imported into the nucleus. Interestingly, immunoprecipitation analysis indicated that TMX2 interacted with Ran, especially RanQ69L (Fig. 1E). To define the intracellular interaction of TMX2 and importin-β or Ran, bimolecular fluorescence complementation (BiFC) assay was performed (Fig. 1F). The assay is based on the discoveries that two non-fluorescent N- and C-terminal fragments of a fluorescent protein can form a fluorescent complex when they are fused to a pair of interacting proteins^22,23^. We prepared TMX2, importin-β, or Ran fused to the fragment of Venus protein, and confirmed that these fusion proteins do not emit fluorescence by itself (see Supplementary Fig. S2). When TMX2 fused to N-terminal fragment of Venus (VN155, I152L) was co-expressed with importin-β- or Ran-fused C-terminal fragment of Venus (VC155) in HEK293 cells, Venus fluorescence was detected in the nuclear envelope as BiFC signal, and this signal localization was similar with the BiFC signal by co-expression of Ran-VN and importin-β-VC. These results indicate that TMX2 interacts with importin-β and Ran, especially RanQ69L (GTP-bound form), in the nuclear envelope. To demonstrate the localization of TMX2 in the nuclear envelope, emerin, which localizes in the inner membrane of the nuclear envelope, was expressed in HEK293 cells and their co-localization was analyzed. Emerin was fused with Flag tag at the C terminus, and Flag tag was located at the lumen of the nuclear envelope, known as the perinuclear space^24^. The expression pattern of endogenous TMX2 partially overlapped with that of emerin in the nuclear envelope, but it was slightly outside of emerin in the inner membrane, as indicated by the intensity profile (Fig. 1G), suggesting that TMX2 localizes in the outer membrane of the nuclear envelope.

### TMX2 isoform 2, which lacks a part of the transmembrane region, did not localize in the nuclear envelope

We found that in addition to the TMX2 isoform 1 (NP_057043.1), the mRNA of TMX2 isoform 2, which encodes a protein (NP_001137484.1) (258 amino acids) with deletion of amino acids 84–122 of isoform 1 (WT), was expressed in HEK293 cells. However, the levels of protein and mRNA of TMX2 isoform 2 were relatively less abundant than those of isoform 1 (Fig. 1C,I). Isoform 2 was isolated and the Venus-fused TMX2 isoform 2 was expressed in HEK293 cells. While TMX2 WT-Venus localized in the nuclear envelope and the ER, TMX2 isoform 2 localized in the ER, but not in the nuclear envelope and did not co-localize with emerin (Fig. 1J). Isoform 2 lacks a main transmembrane region of isoform 1 (Fig. 1H), suggesting that this region is required for localization in the nuclear envelope.

### Localization and topology of TMX2 in the nuclear envelope

**Figure 2:**
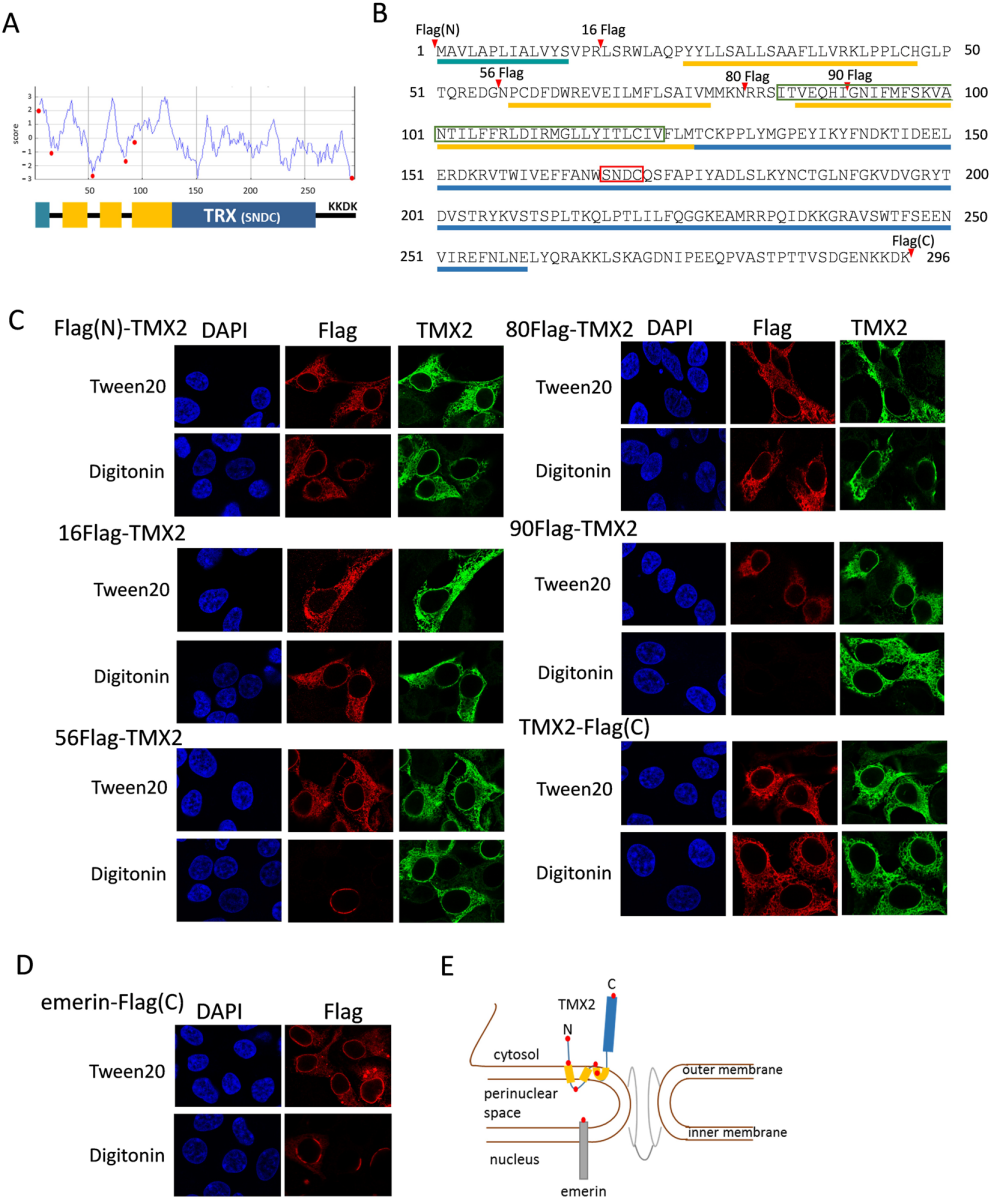
Topology of TMX2 in the nuclear envelope. (**A**) A hydrophobic profile of the TMX2 protein was obtained by the SOSUI program, and predicted its transmembrane regions. (**B**) Protein sequences of TMX2. The predicted transmembrane regions are indicated by yellow bars, and thioredoxin-like domain was by blue bar. TMX2 isoform 2 lacks part of the transmembrane region as indicated by green box. The conserved S-X-X-C motif was indicated by red box. (**C**) Flag tag was inserted into TMX2 at the N- or C- terminus, or the amino acid position 16, 56, 80, or 90 as indicated by red arrowheads (**A**) or red circles (**B**), and overexpressed in HEK293 cells. These cells were fixed with PFA, and permeabilized by Tween20 or digitonin. The localizations of these proteins were analyzed with anti-Flag antibody or polyclonal anti-TMX2 antibody, which recognizes an epitope in full length TMX2. (**D**) Flag fused-emerin at the C terminus was expressed in cells, and permeabilized by Tween20 or digitonin after fixation. (**E**) The speculated topology of TMX2 in the nuclear outer membrane.

The membrane-spanning region of protein is typically comprised of 20–30 amino acid residues as an α-helix, and the hydrophobic profile of the TMX2 protein obtained by the SOSUI program suggested that it has several transmembrane regions (Fig. 2A,B). To investigate the topology of TMX2 in the nuclear envelope, Flag tag was inserted into TMX2 at the N- or C- terminus, or at amino acid position 16, 56, 80, or 90 as indicated by the red circles in Fig. 2A,B, and the Flag tag signal was detected by immunofluorescence (Fig. 2C). Digitonin was used as a detergent that only permeabilizes the plasma membrane at a concentration of 40 μg/mL, but that also has been shown to partially permeabilize the outer nuclear membrane and leave the inner nuclear membrane intact^24^, while Tween20 permeabilizes all cellular membranes. Insertion of Flag tag into any position of TMX2 did not affect its localization in the nuclear envelope as indicated by the signal of Flag after permeabilization by Tween20. The fluorescence signals of all Flag tag-inserted TMX2 proteins were detectable by polyclonal anti-TMX2 antibody, which recognizes an epitope in full-length TMX2, even with digitonin treatment, suggesting that part of TMX2 faces the cytoplasm. By permeabilization with digitonin, the signal for Flag tag was detected in Flag(N)-TMX2, TMX2-Flag(C), 16Flag-TMX2, or 80Flag-TMX2, suggesting that the Flag tags within these TMX2 proteins face the cytoplasm. However, 56Flag-TMX2 was not detected, or was detected only partially in the nuclear envelope by anti-Flag antibody with digitonin. We confirmed that digitonin can partially permeabilize the outer membrane of the nuclear envelope, not only the plasma membrane, based on the result that the signal of Flag tag which faces the perinuclear space in emerin-Flag protein was not detected, or detected only partially in the nuclear envelope (Fig. 2D). These results indicate that the Flag tag at amino acid position 56 in TMX2 was located in the perinuclear space. On the other hand, Flag-tag signaling at 90Flag-TMX2 was not detected, raising the possibility that the Flag tag was buried in the outer nuclear envelope. From these results we speculated the topology of TMX2 in the nuclear envelope, as illustrated in Fig. 2E.

### Binding of TMX2 with importin-β and Ran

**Figure 3:**
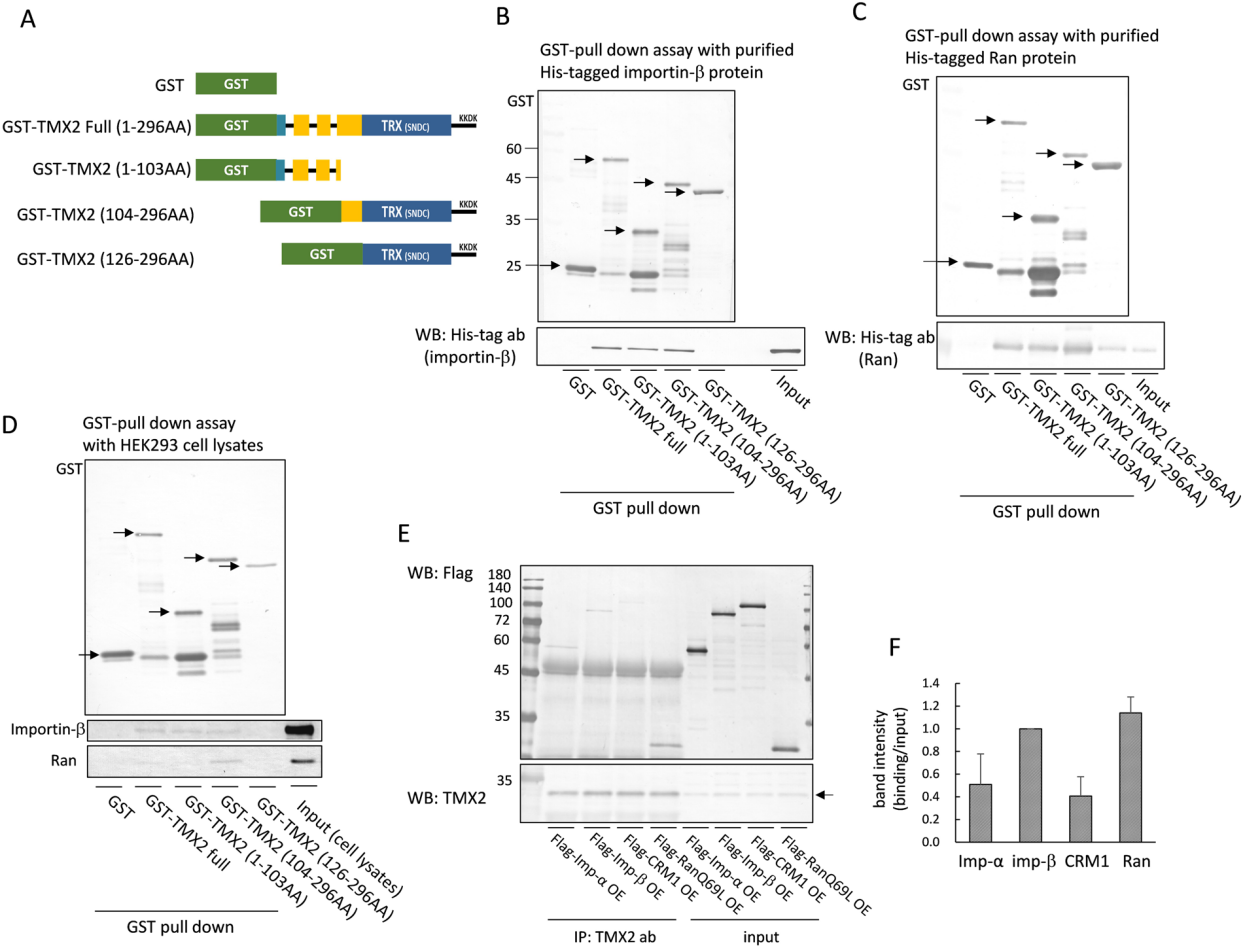
Binding between TMX2 and importin-β or Ran. (**A**) Scheme of GST-tagged TMX2 full-length and deletion mutants. (**B**,**C**) GST-tagged TMX2 proteins were immobilized on glutathione-Sepharose beads and incubated with purified His-tagged importin-β or Ran protein. The precipitated importin-β or Ran was analyzed by immunoblot with anti-His tag antibody. (**D**) TMX2-packed glutathione-Sepharose was incubated with HEK293 cell lysates, and the precipitant with TMX2 was analyzed with anti-importin-β or Ran antibody. (**E**) Flag-importin-α, -importin-β, -CRM1, or -RanQ69L was expressed in HEK293 cells, and cell lysates were immunoprecipitated with anti-TMX2 antibody. The precipitates were detected with anti-Flag antibody. (**F**) The ratio of band intensity of binding/input was quantitated. Values are the means ± S.D. for three separate experiments. The value of importin-β was set at 1.0.

To investigate the binding region of TMX2 with importin-β and Ran, GST-tagged TMX2 (full length, 1–103 AA, 104–296 AA, or 126–296 AA, as indicated in Fig. 3A) was immobilized on glutathione-Sepharose beads and incubated with purified His-tagged importin-β or Ran protein (Fig. 3B,C) or HEK293 cell lysates (Fig. 3D). An *in vitro* binding assay with purified proteins indicated that TMX2 can directly bind to importin-β and Ran (Fig. 3B,C). Importin-β bound to both the N-terminal (1–130 AA) and C-terminal (104–296) region of TMX2, but not to the C-terminal region (126–296 AA), which lacks a main transmembrane region (Fig. 3B,D). Purified Ran protein also bound to both the N-terminal and C-terminal region of TMX2, but the binding with the C-terminal region was stronger than that with the N-terminal region (Fig. 3C). Similarly, cellular Ran protein strongly bound to the C-terminal region of TMX2 (Fig. 3D). Ran also required the main transmembrane region of TMX2, 104–125 AA, for its binding with the C-terminal region of TMX2. To investigate the specificity of the binding of TMX2 to importin-β and Ran, we compared their binding with that to importin-α, and CRM1. Flag-importin-α, -importin-β, -CRM1, or -RanQ69L was expressed in HEK293 cells, and the binding between these Flag-tagged proteins and endogenous TMX2 was investigated by immunoprecipitation. Importin-α and CRM1 were co-precipitated with TMX2 (Fig. 3E), but the ratio of the precipitate to the input signal intensity for importin-α and CRM1 was lower than that for importin-β and Ran (Fig. 3F), suggesting that TMX2 preferentially binds with importin-β and Ran. To pass through nuclear pore complexes, importin-α, which recognizes the classical NLS of protein cargoes, is imported with importin-β from the cytosol to the nucleus, while the exportin CRM1, which recognizes the nuclear export signal (NES) of protein cargoes, is exported with Ran GTP from the nucleus to the cytosol. Therefore, it is possible that importin-α and CRM1 indirectly bind to TMX2 via importin-β and Ran.

### TMX2 regulates the nucleocytoplasmic Ran protein gradient

**Figure 4:**
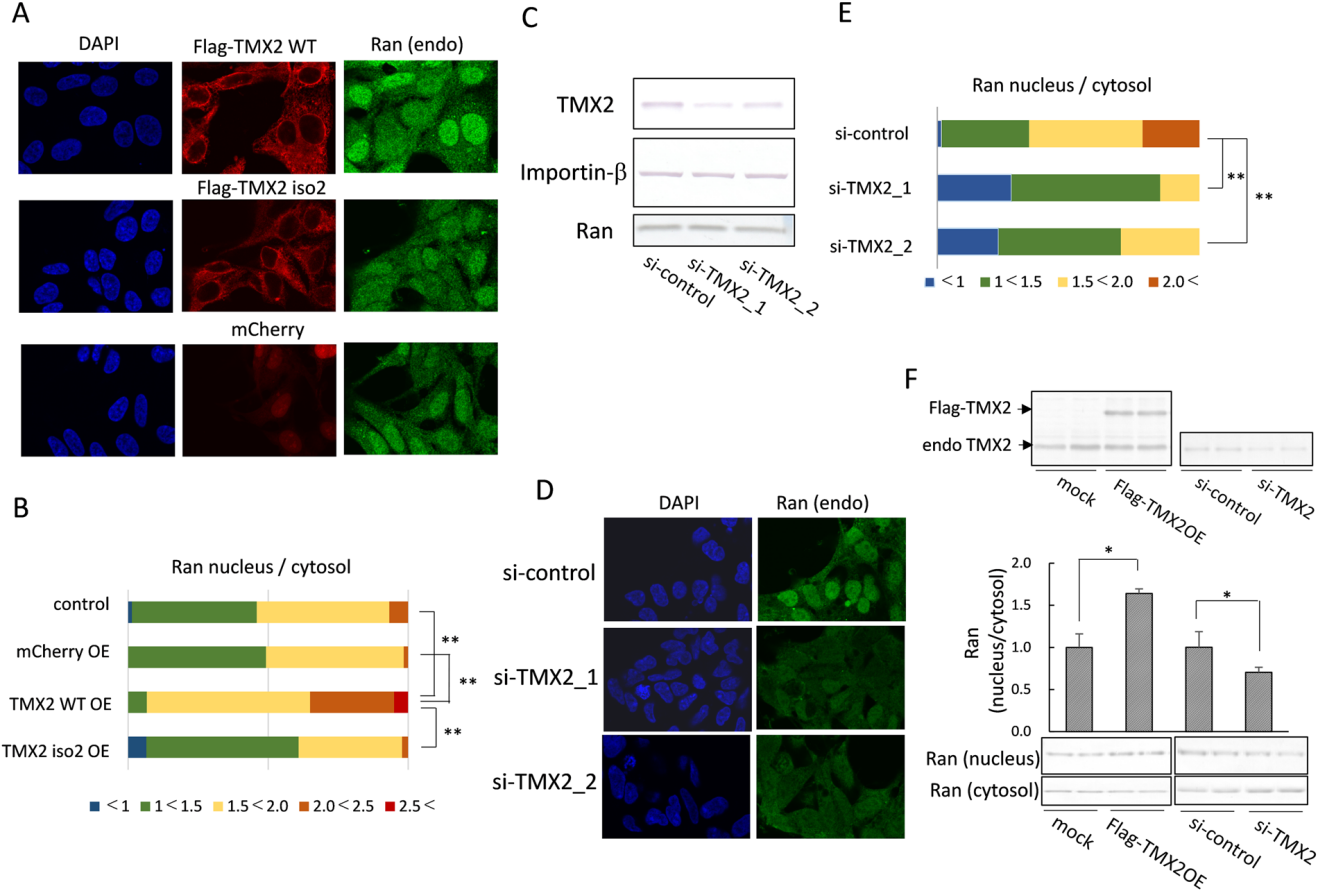
Regulation of the Ran protein gradient by TMX2. (**A**) Flag-TMX2 WT, Flag-isoform 2, or mCherry was overexpressed in HEK293 cells, and the distribution of endogenous Ran was analyzed by immunofluorescence microcopy. (**B**) The ratio of Ran levels in the nucleus/cytosol of each kind of cell (n = 60) was quantified. (**C**) si-RNA targeting for the negative control or TMX2 was lipofected into HEK293 cells, and TMX2, importin-β, or Ran protein levels were analyzed by western blotting. (**D**) Endogenous Ran distribution in TMX2 knockdown cells was analyzed by immunofluorescence microcopy. (**E**) The ratio of Ran levels in the nucleus/cytosol of each kind of cell (n = 60) was quantified. (**F**) The nuclear and cytosolic fractions of TMX2-overexpressing or -knockdown cells were extracted, and analyzed by western blotting with anti-Ran antibody. Values are the means ± S.D. for three experiments. The Ran nucleus/cytosol ratio of control cells (mock or si-control) was set at 1.0. *p < 0.05, **p < 0.01.

The Ran protein predominantly localized in the nucleus, and small amounts were also present in the cytoplasm. Maintaining the Ran protein gradient between the cytoplasm and nucleoplasm is essential to driving the nucleocytoplasmic cargo transport. To investigate the function of TMX2 in Ran localization, TMX2 was overexpressed in HEK293 cells. The nuclear Ran levels were increased by overexpression of the TMX2 WT compared with non-transfected cells, while TMX2 isoform 2 did not affect Ran distribution (Fig. 4A). Quantification of the Ran signal intensity shows that the nucleus/cytosol ratio for Ran distribution in TMX2 WT-overexpressed cells was higher than that in non-transfected cells (control) or mCherry-overexpressed cells (Fig. 4B), indicating that TMX2 in the nuclear envelope facilitates the Ran gradient. On the other hand, knockdown of TMX2 by si-RNA suppressed nuclear Ran levels (Fig. 4D,E), although TMX2 knockdown did not decrease total Ran levels (Fig. 4C). Quantification of the Ran nucleus/cytosol signal-intensity ratio indicated that TMX2 knockdown shifts the Ran distribution from the nucleus to the cytosol (Fig. 4E). These results suggest that endogenous TMX2 is important in the maintenance of the nucleocytoplasmic Ran protein gradient. The same results were also obtained by the experiment of nuclear extraction in HEK293 cells by overexpression or knockdown of TMX2 (Fig. 4F).

### Function of TMX2 in the importin-β-dependent transport of cargo proteins

**Figure 5:**
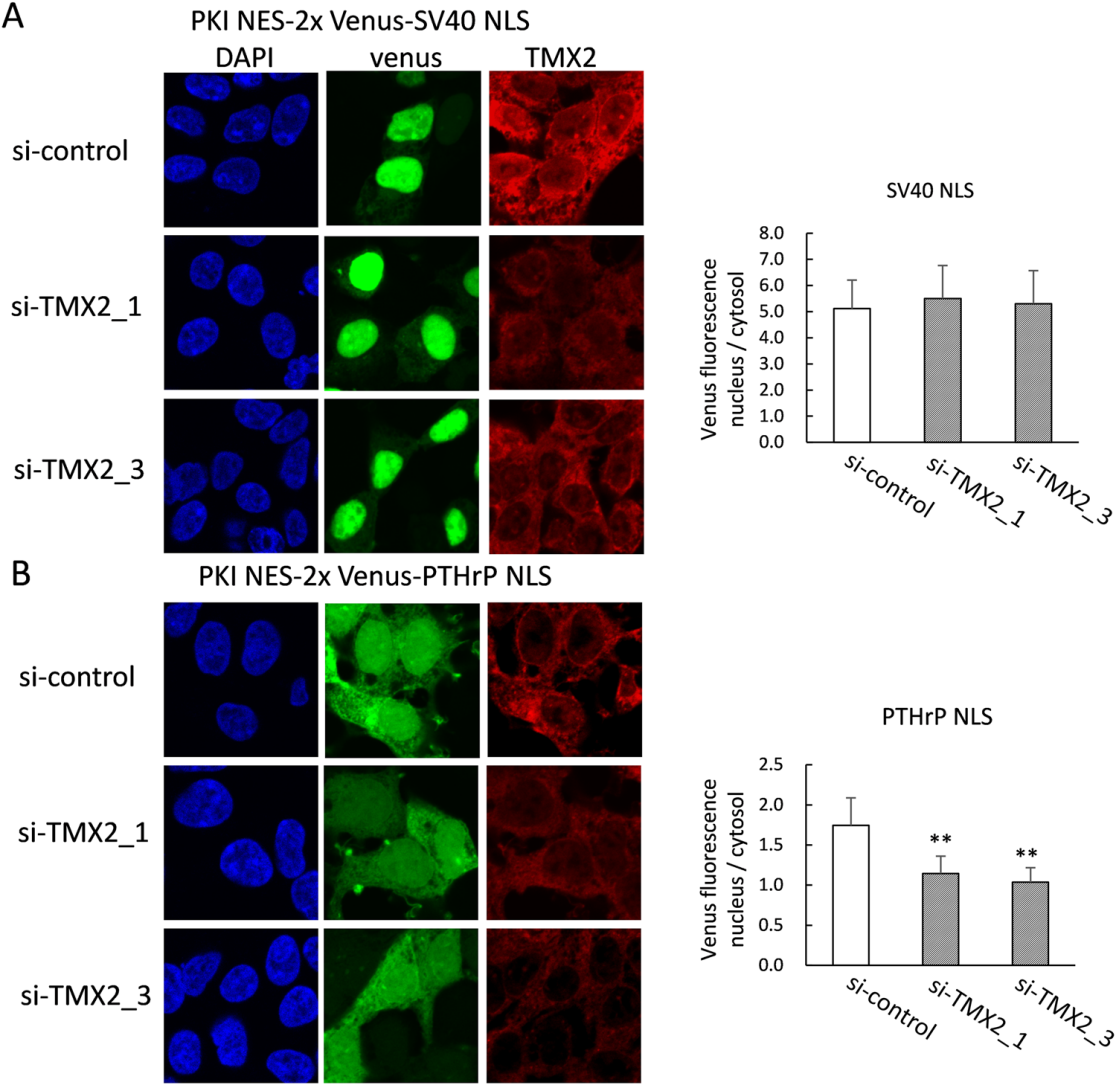
TMX2 knockdown decreased importin-β-dependent nuclear transport of protein. The expression plasmid for NES-2xVenus-SV40 NLS (**A**) or NES-2xVenus-PTHrP NLS (**B**) was co-transfected with control si-RNA or si-TMX2 into HEK293 cells. After 48 h, cells were fixed and incubated with anti-TMX2 antibody, followed by secondary antibody labeling with Dylight 647. The nucleus/cytosol ratio of Venus fluorescence was measured in about 30 cells. Values are the means ± S.D. **p < 0.01 compared with the si-control.

Disruption of the Ran gradient has been shown to reduce the activity of the importin-β-dependent cargo transport system. Based on the disruption of the Ran gradient by TMX2 knockdown, we next investigated the effect of TMX2 knockdown on importin-mediated protein transport to the nucleus. Protein cargoes with classical NLS such as SV40 are imported into the nucleus by importin-α/β, while some cargoes with NLS are imported only by importin-β. PTHrP has a long non-classical NLS which is recognized directly by importin-β, independent of importin-α^25^. A 2xVenus protein with NES of human protein kinase inhibitor (PKI) and either an importin-α/β-dependent classical NLS of SV40, or an importin-β-dependent NLS of PTHrP was expressed with si-RNA targeting TMX2. Although knockdown of TMX2 did not affect transport of the cargo protein with SV40 NLS (Fig. 5A), it suppressed the nucleus/cytosol ratio of proteins with the PTHrP NLS (Fig. 5B), suggesting that TMX2 contributes to the transport of importin-β-dependent cargo proteins into the nucleus by maintaining the Ran protein gradient.

### The cysteine 112 of Ran was involved in the regulation of Ran gradient by TMX2

**Figure 6:**
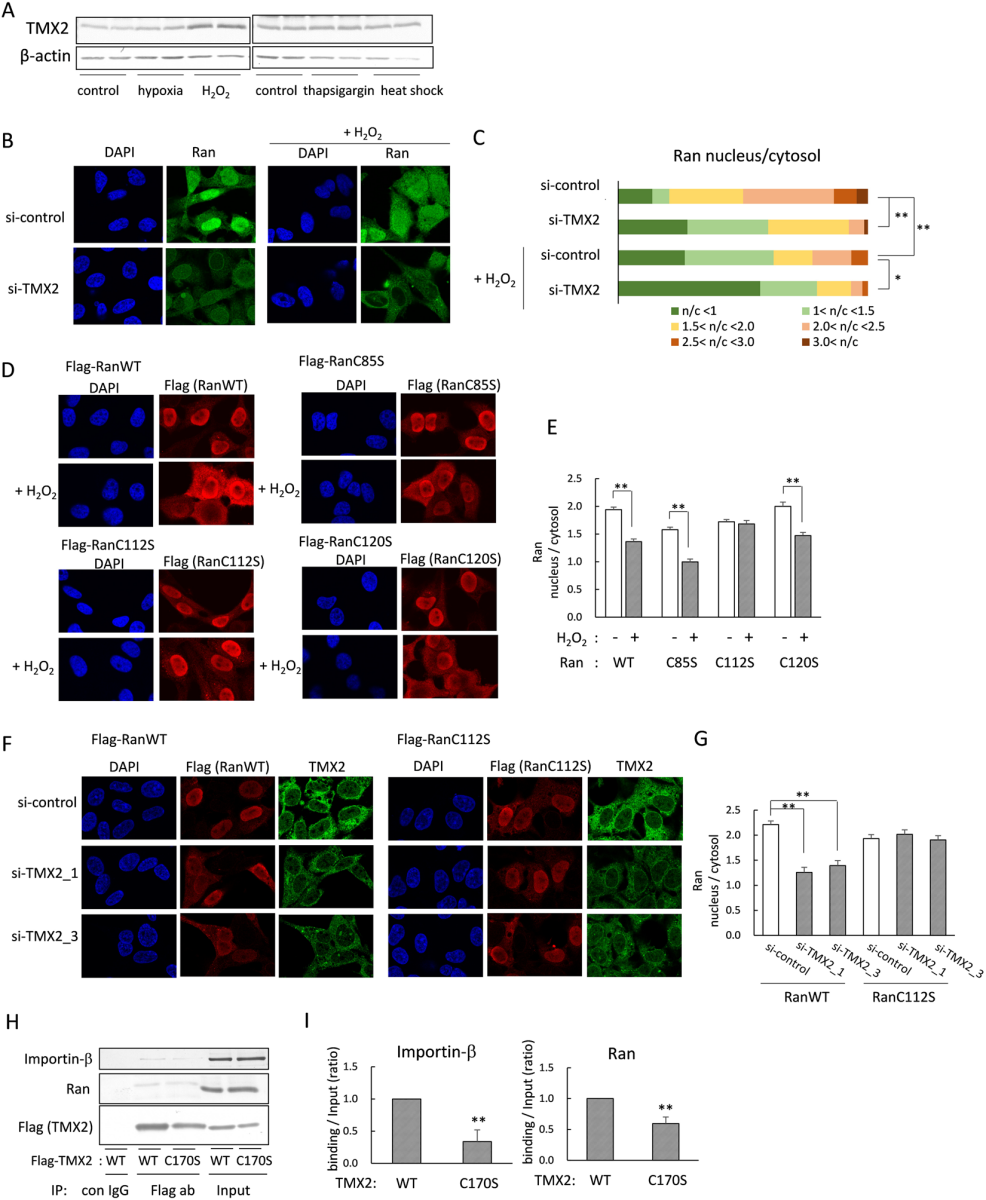
The cysteine 112 of Ran was involved in the regulation of Ran gradient by TMX2. (**A**) HEK293 cells were cultured under 1% oxygen concentration for 6 h or heat stress condition at 42 °C for 3 h, or in the presence of 200 μM H_2_O_2_ for 4 h or 200 nM thapsigargin for 4 h, and TMX2 protein induction was investigated. (**B**) Hela cells were lipofected with si-control or si-TMX2, and treated with 200 μM H_2_O_2_ for 20 min. After fixing with PFA, the endogenous Ran distribution was observed by immunofluorescence microscopy. (**C**) The Ran nucleus/cytosol signal-intensity ratios of about 40 cells were quantitated. (**D**) Flag-Ran WT, C85S, C112S, or C120S was expressed in Hela cells, and the cells were treated with 200 μM H_2_O_2_ for 30 min. The localization of Ran was analyzed by immunostaining with anti-Flag antibody. (**E**) The nucleus/cytosol ratio of Ran was quantified in about 45 cells. Values are the means ± S.E. (**F**) Ran WT or C112S/Flag pcDNA was co-transfected with si-TMX2 into cells. After 48 h, the localization of Ran was analyzed by anti-Flag antibody. Knockdown of TMX2 was confirmed by anti-TMX2 antibody. (**G**) The nucleus/cytosol ratio of Ran was quantified in about 40 cells. (**H**) HEK293 cells were overexpressed with Flag-tagged TMX2 WT or C170S mutant, and the cell lysates were immunoprecipitated with anti-Flag antibody. The precipitant was analyzed by anti-importin-β or Ran antibody. (**I**) The band intensity of importin-β or Ran binding/Input was quantified. Values are the means ± S.D. for three separate experiments. The control value was set at 1.0. *p < 0.05, **p < 0.01.

Nuclear transport of proteins is altered by several cellular stressors such as heat or oxidative stress^26,27^. Because PDI family proteins are known to be induced by various stress conditions, we examined which stress conditions induce TMX2 levels. We cultured HEK293 cells under 1% hypoxia for 6 h, 200 μM H_2_O_2_ for 4 h, 200 nM thapsigargin as an ER stressor for 4 h, or heat stress at 42 °C for 3 h. The results showed that TMX2 was induced by oxidative stress, but not by hypoxic, ER, or heat stress (Fig. 6A). It was previously shown that oxidative stress by treatment of H_2_O_2_ alters distribution of Ran from the nucleus to the cytosol in Hela cells^28^. We also confirmed that treatment with 200 μM H_2_O_2_ for 20 min suppressed nuclear Ran levels in Hela cells, and that TMX2 knockdown additionally suppressed nuclear Ran levels in the presence of H_2_O_2_ (Fig. 6B). Quantification of the Ran nucleus/cytosol signal-intensity ratio indicated that Ran was predominantly distributed in the cytosol in more than half of the H_2_O_2_-treated TMX2 knockdown cells (Fig. 6C). The significant effects of H_2_O_2_ on the nucleocytoplasmic Ran protein gradient in TMX2 knockdown cells suggest that TMX2 has the role of protecting cells from oxidative stress by maintain a Ran gradient, and the induction of TMX2 by oxidative stress may be a protective response of cells to the stress. These results raise a possibility that TMX2 maintains Ran gradient by changing a redox state of Ran. Ran possesses three cysteine residues C85, C112, C120. Each cysteine was substituted by serine, and a sensitivity of these mutants to H_2_O_2_ was investigated (Fig. 6D,E). The substitution of cysteine residues did not affect their distribution under normal condition; predominantly localized in the nucleus as well as WT. By the treatment of H_2_O_2_, however, the nuclear expression of WT and C85S or C120S mutant were decreased, while C112S mutant was not affected and abundantly localized in the nucleus. These results indicate that Cysteine 112 was involved in the disruption of Ran gradient by H_2_O_2_. Then, the distribution of Ran C112S by knockdown of TMX2 was also investigated by co-transfection of Ran/Flag pcDNA with si-TMX2. As a result, nuclear levels of Ran WT was decreased, but the C112S mutant was still kept in the nucleus by knockdown of TMX2. These results suggest that TMX2 may regulate Ran gradient via changing the redox state of cysteine 112 residue of Ran. TMX2 has only a single cysteine 170 in the SNDC motif instead of the thioredoxin CXXC motif. Although ERp44 and ERp29 also do not conserve CXXC, and have only a single cysteine, the single cysteine has been shown to be important for their binding with their interacting proteins or redox regulation by collaboration with other redox factors^29,30^. A C170S mutation to TMX2 diminished the binding of TMX2 to importin-β and Ran, although it was not complete (Fig. 6H,I).

## Discussion

In the present study, we found that TMX2 interacted with importin-β and Ran but was not a cargo transported by these proteins. Generally, importin-β imports cargo proteins into the nucleus primarily in complex with the adaptor protein importin-α, which recognizes the classical NLS of cargoes, while CRM1 recognizes the NES of cargoes, and transports them into the cytoplasm with RanGTP. Despite the fact that TMX2 was not transported by these proteins, the classical NLS sequence K-K/R-X-K/R and the NES sequence L-XXX-L-XX-L-X-L were found at 237–240 AA (KKGR), and 213–222 AA (LTKQLPTLIL), respectively, in the TMX2 sequence. We have demonstrated that the localization of TMX2 was not affected by the insertion of a mutation into the sequence of NLS or NES of TMX2 (see Supplementary Fig. S3) or by treatment with Leptomycin B (see Supplementary Fig. S1), and these results also strongly indicate that TMX2 is not an import or export cargo of importin-α and CRM1. Immunoprecipitation analysis indicated that endogenous TMX2 preferentially interacts with importin-β and Ran, but also interacts weakly with importin-α and CRM1. In addition, we found that the binding strength of overexpressed TMX2 with importin-α or CRM1 was comparable to that with importin-β or Ran (see Supplementary Fig. S4). Though it has not been revealed whether the binding between TMX2 and importin-α or CRM1 was direct or indirect, elucidating the role of these signal sequences in TMX2 may reveal an interesting function of TMX2.

TMX2 was suggested to be an integral membrane protein located at the outer nuclear membrane, and interacted with importin-β and Ran. Both the N-terminus and C-terminus of TMX2 face the cytoplasm, suggesting that TMX2 interacts with these transport proteins at the cytoplasmic surface of the nuclear envelope. TMX2 has an ER-retention signal, KKDK, in its C-terminus, and is abundantly expressed in the ER as well as in the outer nuclear membrane. Though the outer nuclear membrane is contiguous with the ER membrane, it has been shown that several integral membrane proteins such as Nesprin are enriched at the outer nuclear membrane by interaction with inner nuclear membrane proteins spanning the perinuclear space^31^. Hence, TMX2 may also be tethered by other proteins in the perinuclear space. The fact that TMX2 isoform 2, which lacks amino acids 84–122 containing the main transmembrane region of WT, did not show distribution in the nuclear envelope, suggest that this region is necessary for interactions with the tethering proteins.

We found that localization of TMX2 in the nuclear envelope was important to maintaining the nucleocytoplasmic Ran protein gradient. Ran is predominantly located in the nucleus, and most nuclear Ran is in the GTP-bound form due to the activity of nucleotide exchange factor (RCC1), which replaces the GDP bound to Ran with GTP^32^. The binding of RanGTP with importin-β in the nucleus is necessary to release cargo from importin-β, and the importin-β-RanGTP complex is transported from the nucleus to the cytosol for recycling of the importin-β. The importin-β-RanGTP complex dissociates by conversion of RanGTP into RanGDP, which is stimulated by RanGAP and RanBP2 at the nuclear core complex in the cytoplasm because the intrinsic GTP hydrolysis rate of Ran is very slow^33^. RanGAP binds to RanBP2, which is a component of the cytoplasmic fibrils of the nuclear pore complex; they aid in RanGTP hydrolysis and disassembly of transport receptors such as importin-β-Ran complex^34,35^. RanBP2 has SUMO E3 ligase activity, and sumoylates the hydrolyzed RanGDP, which is suggested to allow the import of Ran into the nucleus by Nuclear transport factor 2 (NTF2)^35^. Thus, the interaction between the importin-β-RanGTP complex and RanGAP-RanBP2 is necessary for the recycling pathway of importin-β and Ran for the next round of transport. BecauseTMX2 preferentially interacted with RanQ69L (the GTP-bound form), and RanGAP and RanBP2 have also been identified as TMX2-interacting proteins as well as importin-β and Ran, TMX2 may facilitate each event or series of events: namely, GTP hydrolysis of Ran, importin-β-Ran disassembly and the sumoylation of Ran on TMX2 in the cytoplasmic surface of the nuclear envelope. Consequentially, TMX2 will maintain the Ran gradient by driving the reentry of Ran into the nucleus by NTF2, and also accelerates the recycling of importin-β for the import of the cargo into the nucleus.

Indeed, TMX2 was important for maintaining the importin-β-dependent cargo import. However, TMX2 knockdown inhibited the transport of cargo with PTHrP NLS, which is directly recognized by importin-β, but not the classical NLS of SV40, which is imported by importin-α/β. Many reports have indicated that disruption of the Ran gradient leads to impairment of importin-β-dependent cargo import, but it has been shown that disruption of the Ran gradient in Hutchinson-Gilford Progeria syndrome (HGPS) fibroblasts did not affect transport of the classical SV40 NLS-cargo protein, despite its alteration of the transport of the Tpr protein, which has bipartite NLS^21,36^. These findings suggest that the efficiency of the nuclear import of these cargoes reflects the Ran sensitivity of the transport pathway.

We found that TMX2 was also not induced by hypoxic, heat, or ER stress, similarly to the finding that TMX1^6^, TMX3^4^, and TMX4^5^ are not induced by heat or ER stress; however, oxidative stress by H_2_O_2_ treatment did induce TMX2 expression. It was previously found that H_2_O_2_ treatment alters the Ran distribution from the nucleus to the cytosol^28,37^. We confirmed this phenomenon, and treatment of cells with oxidizing agent, diamide, or hypoxia-reoxygenation also decreased nuclear Ran levels (see Supplementary Fig. S5). Intracellular GTP depletion accompanying a decrease in ATP levels^28^, or activation of ERK2^38^ by oxidative stress has been suggested to be involved in the alteration of Ran distribution, but the mechanism is not fully understood. In the present study, we found that Cys112 of Ran was involved in the alteration of Ran distribution by hydrogen peroxide or TMX2 knockdown. The Cys112 of Ran has been shown to exposed on the surface of the Ran molecule, and could be oxidized^39^. The oxidation of the Cys112 may disturb the entry of Ran into the nucleus, and TMX2 will contribute to keep the cysteine in its reduced state. Further study is necessary to elucidate the redox change of Ran and subsequent alteration of its function including interaction with nuclear pore complex, and GTP hydrolysis. We also suggest that the induction of TMX2 by oxidative stress is a response to mitigate the effects of the oxidative stress. Although TMX2 itself does not have oxidoreductase activity as a PDI family protein, the single conserved cysteine 170 in the thioredoxin motif of TMX2 was important for the binding with importin-β and Ran, but was not essential. It has been shown that a PDI family member, ERp44, which does not have a CXXC thioredoxin motif, has the capability of forming mixed disulfides with its clients via the cysteine in its conserved active site, the CXXS motif, and regulates oxidoreductase state of the client protein in collaboration with the redox factor, ERO1^40,41^. To clarify the redox partner of TMX2 and the redox regulation of Ran by TMX2 will reveal an interesting mechanism for the nuclear transport system.

## Materials and Methods

Dulbecco’s modified Eagle’s medium (DMEM), DMEM/Ham’sF-12, anti-DYKDDDDK (Flag) antibody and anti-GFP(VC) antibody (mFX75) were purchased from Fujifilm Wako Pure Chemical Corporation (Osaka, Japan). Fetal bovine serum (FBS), penicillin–streptomycin solution, and digitonin were purchased from Sigma Chemical (St. Louis, MO), DAPI solution from Dojindo (Kumamoto, Japan), anti-Ran antibody from Bethyl Laboratories Inc. (Montgomery, TX), anti-HA antibody (M180-3) from MBL (Nagoya, Japan), and protein A Sepharose and glutathione Sepharose 4B from GE Healthcare (Chicago, IL).

### Preparation of constructs

The entire coding regions of human TMX2 WT (isoform1) (Accession No. NM_015959.4) and TMX2 isoform 2 (Accession No. NM_001144012.2) were amplified from Hep3B cDNA with the primers 5′- AAGGATCCGAAAAGATGGCGGTCTTG -3′ and 5′- AAGAATTCTTATTTATCCTTCTTGTTTTCCCCA -3′, and inserted into 3xFlag-pcDNA4 with BamHI and EcoRI. For TMX2-Venus constructs, the Venus sequence was inserted into pcDNA3.1( + ) with NotI and XbaI, and cDNA of TMX2 WT or isoform 2 without the stop codon was inserted with BamHI and EcoRI. TMX2 WT cDNA was also inserted into a pET21a vector or pGEX-5 × -1 vector for protein expression in *E. coli*. A TMX2 cDNA-encoding region (1-103AA, 104–296AA, or 126–296AA) was inserted into a pGEX-5 × -1 vector. For analysis of the topology of TMX2, a 1xFlag tag was inserted at the N- or C-terminus, or at amino acid position 16, 56, 80, or 90 of TMX2 in pcDNA3.1( + ). For BiFC assay, TMX2 cDNA without the stop codon was inserted into pBiFC-VN155(I152L) vector (Addgene, Watertown, MA). For preparation of the importin-β-mCherry construct, human importin-β was amplified with the primers 5′- AAGGATCCTCCGCCATGGAGCTGATC -3′ and 5′-TTATTATTGCGGCCGCCAGCTTGGTTCTTCAGTTTCCTCAGT-3′, and inserted with BamHI and NotI into pcDNA3.1( + ), in which mCherry sequence had been inserted with NotI and XbaI. For preparation of importin-β-VC155 construct, linker + VC155 sequence was amplified from pBiFC-VC155 vector (Addgene) and inserted into the above vector with NotI and XbaI, instead of mCherry sequence. Importin-β cDNA was also inserted into 3xFlag-pcDNA4 and pQE80L vector. For the emerin-Flag construct, a 3xFlag tag sequence was inserted into pcDNA with NotI and XbaI; then, human emerin cDNA was amplified with the primers 5′-AAGGATCCCCCGCCATGGACAACTACGCAGATCTTTC-3′ and 5′- ATTATTATGCGGCCGCCGAAGGGGTTGCCTTCTTCAGCCTGC-3′, and inserted into the 3xFlag-pcDNA with BamHI and NotI. Human Ran cDNA was amplified with the primers 5′-AAGGATCCATGGCTGCGCAGGGAGAGCCCC-3′ and 5′-TTTCTCGAGTCACAGGTCATCATCCTCATCCGGG-3′ and inserted into 3xFlag-pcDNA4 with BamHI and XhoI. Ran cDNA was also inserted into pQE80L for protein expression in *E. coli*, and into pBiFC-VC155 or pBiFC-VN155(I152L) vector. Human importin-α cDNA was amplified with the primers 5′-AAGGATCCATGTCCACCAACGAGAATGCTAA-3′ and 5′-TTGAATTCCTAAAAGTTAAAGGTCCCAGGAG-3′, and inserted into 3xFlag-pcDNA4 with BamHI and EcoRI. Human CRM1 cDNA was amplified with 5′-AAGGATCCTCTATGCCAGCAATTATGAC-3′ and 5′-ATAATAATGCGGCCGCTTAATCACACATTTCTTCTGGAA-3′, and inserted into 3xFlag-pcDNA4 with BamHI and NotI. For preparation of NES-2xVenus-NLS, the Venus sequence without the stop codon was inserted into pcDNA with HindIII and BamHI, and BamHI and EcoRI. The oligonucleotides for SV40 NLS with the stop codon, 5′-GGCCGCGCTCACCTAAGAAGAAGAGGAAGGTTGAATAAT-3′ and 5′-CTAGATTATTCAACCTTCCTCTTCTTCTTAGGTGAGCGC-3′, were annealed and inserted into 2xVenus/pcDNA with NotI and XbaI. Human PTHrP cDNA encoding amino acids 102–130 containing the NLS was amplified with the primers 5′-AATAATAAGCGGCCGCAAAGATACCTAACTCAGGAAACTAACA-3′ and 5′-AATCTAGATTAGGGCTTGCCTTTCTTTTTC-3′, and inserted into 2xVenus/pcDNA with NotI and XbaI. Then, the oligonucleotides for NES of human cAMP-dependent protein kinase inhibitor alpha (PKI), 5′-AGCTTATGAATGAATTAGCCTTGAAATTAGCAGGTCTTGATATCAACAAGACAA-3′ and 5′-AGCTTTGTCTTGTTGATATCAAGACCTGCTAATTTCAAGGCTAATTCATTCATA-3′, were annealed and inserted into 2xVenus-NLS/pcDNA with HindIII.

### Cell culture and treatment

Hela cells were cultured in DMEM containing 10% (v/v) FBS, 100 units/ml penicillin and 100 μg/ml streptomycin, and HEK293 cells were cultured in DMEM/Ham’sF-12 with 10% FBS and penicillin streptomycin. Cells were maintained at 37 °C in 5% CO_2_ and 95% air.

### RNA interference

siRNA against TMX2 was obtained by Qiagen (Hilden, Germany). The target sequence of si-TMX2_1 (cat. No. SI00112735) is CUAGAUUUAACCCUAAGGUAA, that of si-TMX2_2 (cat. No. SI00112742) is AAGGUGGAUGUUGGACGCUAU, and that of si-TMX2_3 (cat.No. SI02777509) is UUCGUUUAUGGUCUUCAUUAA. AllStars Neg. Control (SI03650318, Qiagen) was used as the control siRNA. The siRNA was transfected into the cells with ScreenFect^TM^A Plus (Wako) following the manufacturer’s instructions.

### Immunoprecipitation and nuclear extraction

Cells were washed with PBS and lysed in buffer (50 mM Tris–HCl (pH 7.5) containing 150 mM NaCl and 0.25% NP40) with a protease inhibitor cocktail (Sigma Chemical). Genomic DNA was fragmented by passing the lysed suspension through a needle attached to a syringe, and stored on ice for 30 min. After centrifugation at 14,000 × g for 15 min, the supernatant was incubated with antibody overnight at 4 °C. Protein A-Sepharose was then added, and the mixture was incubated for 1 h at 4 °C. The immunocomplexes were precipitated and washed with buffer (50 mM Tris–HCl (pH 7.5) containing 150 mM NaCl and 0.05% NP40), then analyzed by immunoblotting. Nuclear extracts were prepared from HEK293 cells as described previously^42^.

### Preparation of antibodies

The antibodies against human TMX2 and importin-β were prepared in rabbits using the method described previously^43^. *E. coli*, BL21 (DE3) or DH5α, was transformed with TMX2/pET21a or importin-β/pQE80L, respectively, and the proteins were expressed. The expressed proteins were purified by a Ni-NTA agarose column and used for the preparation of the antibody in rabbits. All experiments were conducted in accordance with guidelines on the welfare of experimental animals and with the approval of the Ethics Committee on the use of animals of Kwansei Gakuin University.

### LC-MS

Protein extracts of HEK293 cells or cells overexpressing Flag-TMX2 were immunoprecipitated with anti-TMX2 or anti-Flag antibody, separated with SDS-PAGE and stained with a SilverQuest™ Silver Staining Kit (Thermo Fisher Scientific, Waltham, MA). Protein bands were excised from the gel and digested in-gel with trypsin. The digested proteins were loaded onto nano-HPLC capillary columns NTCC-360 (75 μm inner diameter, 360 μm outer diameter) containing 15 cm of 3 μm particle-size C18 reverse-phase column material (Nikko Technos, Tokyo) with a gradient of 0 to 40% buffer B (100% acetonitrile and 0.1% formic acid) for 30 min, followed by a gradient of 40 to 100% buffer B for 5 min, and 100% buffer B for 8 min at a flow rate of 300 nL/min (EASY-nLC; Thermo Fisher Scientific). Eluted peptides were analyzed by an LTQ Orbitrap XL mass spectrometer (Thermo Fisher Scientific). The peptide spectral data were searched against the UniProt database using SequestHT.

### Immunofluorescence and BiFC assay

Cells were fixed with 4% paraformaldehyde at 4 °C for 20 min, permeabilized with 0.1% Tween20 and blocked with 0.1% BSA in PBS for 1 h. For digitonin treatment, cells were fixed and incubated with 40 μg/mL digitonin for 3 min, followed by blocking with 0.1% BSA in PBS for 1 h. Then, cells were incubated with specific primary antibodies in blocking solution overnight, and incubated with a secondary antibody, Alexa Fluor 488 goat anti-rabbit IgG (H + L) (Invitrogen, Carlsbad, CA) or anti-mouse IgG (H + L)-Affinity Pure Dylight 594 conjugate (ImmunoReagents Inc., Raleigh, NC) in blocking solution with DAPI for 1 h. Images were obtained by confocal microscopy, Nikon A1 (Nikon, Tokyo) or TCS SP8 (Leica Microsystems, Wetzlar, Germany). For BiFC assay, TMX2 fused to VN155 (N-terminal half of Venus) was co-expressed with importin-β-VC155 (C-terminal half of Venus) or HA-Ran-VC155 in HEK293 cells. Cells were fixed with 4% paraformaldehyde, and the overexpression of each protein was confirmed by anti-TMX2 antibody, and anti-VC or anti-HA antibody, followed by secondary anti-rabbit IgG conjugated with Alexa Fluor 647 and anti-mouse IgG with Dylight 594. Venus fluorescence was detected as BiFC signal.

### *In vitro* binding assay

*E. coli* BL21 was transformed with TMX2 (full-length, 1–103AA, 104–296AA, or 126–296AA)/pGEX-5 × -1, and GST-tagged TMX2 proteins were expressed. *E. coli* was suspended in binding buffer (25 mM Tris–HCl, pH 7.5, containing 75 mM NaCl) and sonicated. The proteins were solubilized by addition of 1% NP40 for 1 h, and the supernatant was collected after centrifugation. Equilibrated glutathione Sepharose 4B was added to the supernatant and incubated for 1 h. The beads were washed with the binding buffer, and the TMX2-packed beads were incubated with HEK293 cell lysates or purified His-tagged importin-β or Ran protein in the binding buffer with 0.25% NP40. The complex was washed and analyzed by SDS-PAGE followed by western blotting.

### Statistical analysis

Statistical analysis for single comparisons between means was carried out with Student’s t test, and *p* values < 0.05 were considered statistically significant. For multiple comparisons, one-way ANOVA followed by a Bonferroni/Dunn post-hoc test was used.

## Supplementary information

Supplementary data

## Data Availability

The datasets generated during and/or analyzed during the current study are available from the corresponding author on reasonable request.

## References

[1] Matsuo Y (2001). Identification of a novel thioredoxin-related transmembrane protein. J Biol Chem.

[2] Lynes EM (2012). Palmitoylated TMX and calnexin target to the mitochondria-associated membrane. EMBO J.

[3] Matsuo Y (2004). TMX, a human transmembrane oxidoreductase of the thioredoxin family: the possible role in disulfide-linked protein folding in the endoplasmic reticulum. Arch Biochem Biophys.

[4] Haugstetter J, Blicher T, Ellgaard L (2005). Identification and characterization of a novel thioredoxin-related transmembrane protein of the endoplasmic reticulum. J Biol Chem.

[5] Sugiura Y (2010). Novel thioredoxin-related transmembrane protein TMX4 has reductase activity. J Biol Chem.

[6] Matsuo Y, Masutani H, Son A, Kizaka-Kondoh S, Yodoi J (2009). Physical and functional interaction of transmembrane thioredoxin-related protein with major histocompatibility complex class I heavy chain: redox-based protein quality control and its potential relevance to immune responses. Mol Biol Cell.

[7] Guerra C, Brambilla Pisoni G, Solda T, Molinari M (2018). The reductase TMX1 contributes to ERAD by preferentially acting on membrane-associated folding-defective polypeptides. Biochem Biophys Res Commun.

[8] Zhao Zhenzhen, Wu Yi, Zhou Junsong, Chen Fengwu, Yang Aizhen, Essex David W. (2019). The transmembrane protein disulfide isomerase TMX1 negatively regulates platelet responses. Blood.

[9] Chao R (2010). A male with unilateral microphthalmia reveals a role for TMX3 in eye development. PLoS One.

[10] Meng X (2003). Cloning and identification of a novel cDNA coding thioredoxin-related transmembrane protein 2. Biochem Genet.

[11] Akiyama M (2014). Identification of UACA, EXOSC9, and TauMuX2 in bovine periosteal cells by mass spectrometry and immunohistochemistry. Anal Bioanal Chem.

[12] Kramer NJ (2018). CRISPR-Cas9 screens in human cells and primary neurons identify modifiers of C9ORF72 dipeptide-repeat-protein toxicity. Nat Genet.

[13] Watanabe S, Harayama M, Kanemura S, Sitia R, Inaba K (2017). Structural basis of pH-dependent client binding by ERp44, a key regulator of protein secretion at the ER-Golgi interface. Proc Natl Acad Sci USA.

[14] Walczak CP, Tsai B (2011). A PDI family network acts distinctly and coordinately with ERp29 to facilitate polyomavirus infection. J Virol.

[15] Terry LJ, Shows EB, Wente SR (2007). Crossing the nuclear envelope: hierarchical regulation of nucleocytoplasmic transport. Science.

[16] Imamoto N (1995). *In vivo* evidence for involvement of a 58 kDa component of nuclear pore-targeting complex in nuclear protein import. EMBO J.

[17] Lott K, Cingolani G (2011). The importin beta binding domain as a master regulator of nucleocytoplasmic transport. Biochim Biophys Acta.

[18] Nagai M, Yoneda Y (2013). Downregulation of the small GTPase ras-related nuclear protein accelerates cellular ageing. Biochim Biophys Acta.

[19] Tsujii A (2015). Retinoblastoma-binding Protein 4-regulated Classical Nuclear Transport Is Involved in Cellular Senescence. J Biol Chem.

[20] Kelley JB (2011). The defective nuclear lamina in Hutchinson-gilford progeria syndrome disrupts the nucleocytoplasmic Ran gradient and inhibits nuclear localization of Ubc9. Mol Cell Biol.

[21] Datta S, Snow CJ, Paschal BM (2014). A pathway linking oxidative stress and the Ran GTPase system in progeria. Mol Biol Cell.

[22] Kerppola TK (2006). Design and implementation of bimolecular fluorescence complementation (BiFC) assays for the visualization of protein interactions in living cells. Nat Protoc.

[23] Kodama Y, Hu CD (2010). An improved bimolecular fluorescence complementation assay with a high signal-to-noise ratio. Biotechniques.

[24] Mamada H, Takahashi N, Taira M (2009). Involvement of an inner nuclear membrane protein, Nemp1, in Xenopus neural development through an interaction with the chromatin protein BAF. Dev Biol.

[25] Lam MH (1999). Importin beta recognizes parathyroid hormone-related protein with high affinity and mediates its nuclear import in the absence of importin alpha. J Biol Chem.

[26] Stochaj U, Rassadi R, Chiu J (2000). Stress-mediated inhibition of the classical nuclear protein import pathway and nuclear accumulation of the small GTPase Gsp1p. FASEB J.

[27] Kodiha M, Chu A, Matusiewicz N, Stochaj U (2004). Multiple mechanisms promote the inhibition of classical nuclear import upon exposure to severe oxidative stress. Cell Death Differ.

[28] Yasuda Y, Miyamoto Y, Saiwaki T, Yoneda Y (2006). Mechanism of the stress-induced collapse of the Ran distribution. Exp Cell Res.

[29] Hisatsune C (2015). ERp44 Exerts Redox-Dependent Control of Blood Pressure at the ER. Mol Cell.

[30] Grumbach Y, Bikard Y, Suaud L, Chanoux RA, Rubenstein RC (2014). ERp29 regulates epithelial sodium channel functional expression by promoting channel cleavage. Am J Physiol Cell Physiol.

[31] Crisp M (2006). Coupling of the nucleus and cytoplasm: role of the LINC complex. J Cell Biol.

[32] Bischoff FR, Ponstingl H (1991). Catalysis of guanine nucleotide exchange on Ran by the mitotic regulator RCC1. Nature.

[33] Klebe C, Bischoff FR, Ponstingl H, Wittinghofer A (1995). Interaction of the nuclear GTP-binding protein Ran with its regulatory proteins RCC1 and RanGAP1. Biochemistry.

[34] Gareau JR, Reverter D, Lima CD (2012). Determinants of small ubiquitin-like modifier 1 (SUMO1) protein specificity, E3 ligase, and SUMO-RanGAP1 binding activities of nucleoporin RanBP2. J Biol Chem.

[35] Sakin V, Richter SM, Hsiao HH, Urlaub H, Melchior F (2015). Sumoylation of the GTPase Ran by the RanBP2 SUMO E3 Ligase Complex. J Biol Chem.

[36] Snow CJ, Dar A, Dutta A, Kehlenbach RH, Paschal BM (2013). Defective nuclear import of Tpr in Progeria reflects the Ran sensitivity of large cargo transport. J Cell Biol.

[37] Miyamoto Y (2004). Cellular stresses induce the nuclear accumulation of importin alpha and cause a conventional nuclear import block. J Cell Biol.

[38] Czubryt MP, Austria JA, Pierce GN (2000). Hydrogen peroxide inhibition of nuclear protein import is mediated by the mitogen-activated protein kinase, ERK2. J Cell Biol.

[39] Tao GZ (2005). Human Ran cysteine 112 oxidation by pervanadate regulates its binding to keratins. J Biol Chem.

[40] Anelli T (2002). ERp44, a novel endoplasmic reticulum folding assistant of the thioredoxin family. EMBO J.

[41] Wang ZV (2007). Secretion of the adipocyte-specific secretory protein adiponectin critically depends on thiol-mediated protein retention. Mol Cell Biol.

[42] Oguro A, Oida S, Imaoka S (2015). Down-regulation of EPHX2 gene transcription by Sp1 under high-glucose conditions. Biochem J.

[43] Ng PS (2003). Production of inhibitory polyclonal antibodies against cytochrome P450s. Drug Metab Pharmacokinet.

