# Supplementary material for: Thioredoxin-related transmembrane protein 2 (TMX2) regulates the Ran protein gradient and importin-β-dependent nuclear cargo transport
Source: Sci Rep. 2019 Oct 25;9:15296. doi: 10.1038/s41598-019-51773-x (PMC6814788; doi:10.1038/s41598-019-51773-x)
Supplement: Supplementary file 1 — Supplementary data [file 41598_2019_51773_MOESM1_ESM.pdf]

Thioredoxin-related transmembrane protein 2 (TMX2) regulates the Ran protein gradient and importin-β-dependent nuclear cargo transport.

Ami Oguro[a,b]*and Susumu Imaoka[a]*

[a]Department of Biomedical Chemistry, School of Science and Technology, Kwansei Gakuin University, Sanda, Japan

[b]Program of Biomedical Science, Graduate School of Integrated Sciences for Life, Hiroshima University, Hiroshima, Japan

*Corresponding author:
Ami Oguro, Ph.D. and Susumu Imaoka, Ph.D.

Department of Biomedical Chemistry, School of Science and Technology, Kwansei Gakuin University,
2-1 Gakuen, Sanda 669-1337, Japan,
Tel/Fax: +81-79-565-7674
E-mail: a.oguro@kwansei.ac.jp

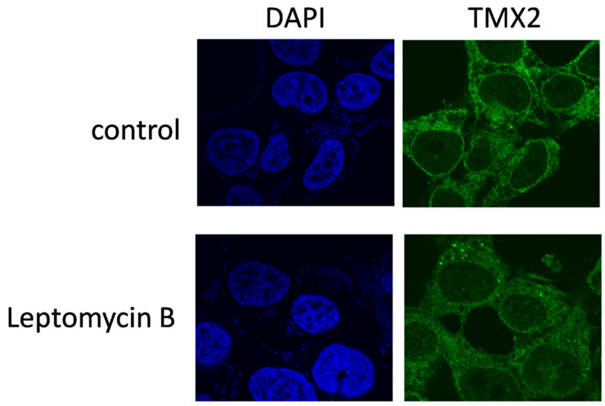

Supplementary Figure S1
TMX2 did not accumulate in the nucleus in the presence of Leptomycin B.
HEK293 cells were treated with 10 nM Leptomycin B for 6 h, and localization of endogenous TMX2 was observed by immunofluorescence microscopy.

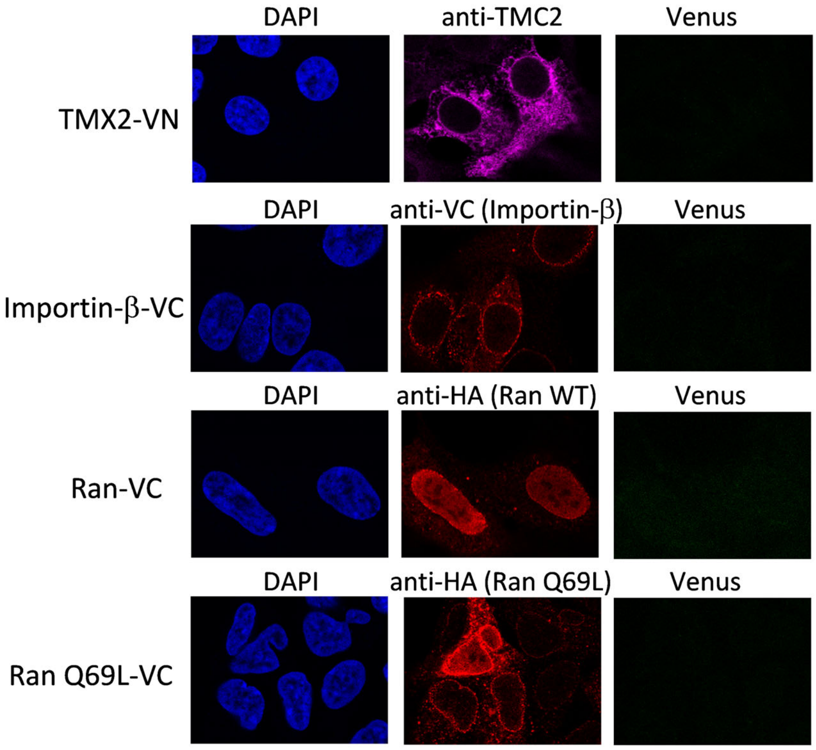

Supplementary Figure S2
VN- or VC- fusion proteins do not emit Venus fluorescence by itself.
TMX2-VN, importin-β-VC, HA-RanWT-VC, or HA-RanQ69L-VC was expressed alone in HEK293 cells, and the overexpression of these proteins were confirmed by anti-TMX2, anti-VC, or anti-HA antibody.

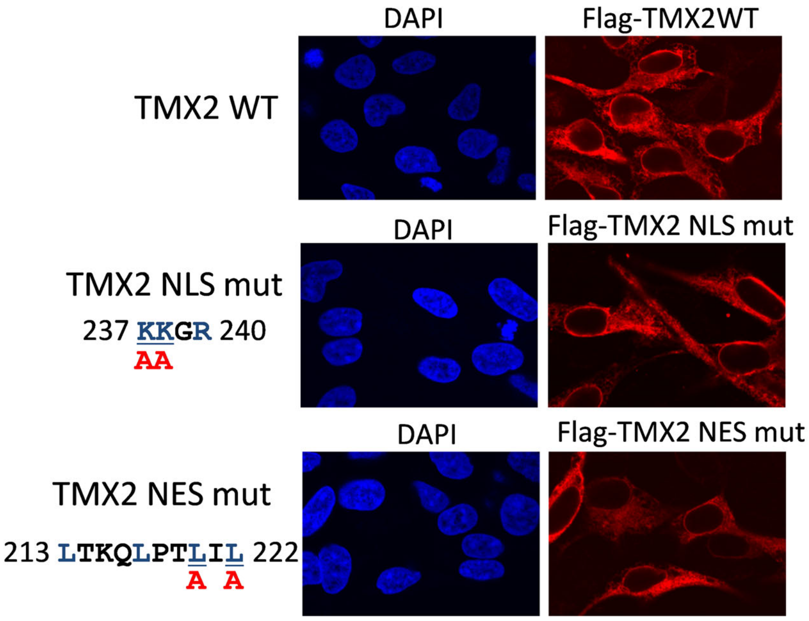

Supplementary Figure S3

The mutation of NLS or NES sequence of TMX2 did not alter the localization of TMX2. TMX2 cDNA with mutation of NLS (K237A and K238A) or NES (L220A and L222A) sequence was inserted into 3xFlag-pcDNA4 vector, and the localization of these proteins were analyzed by immunostaining with anti-Flag antibody.

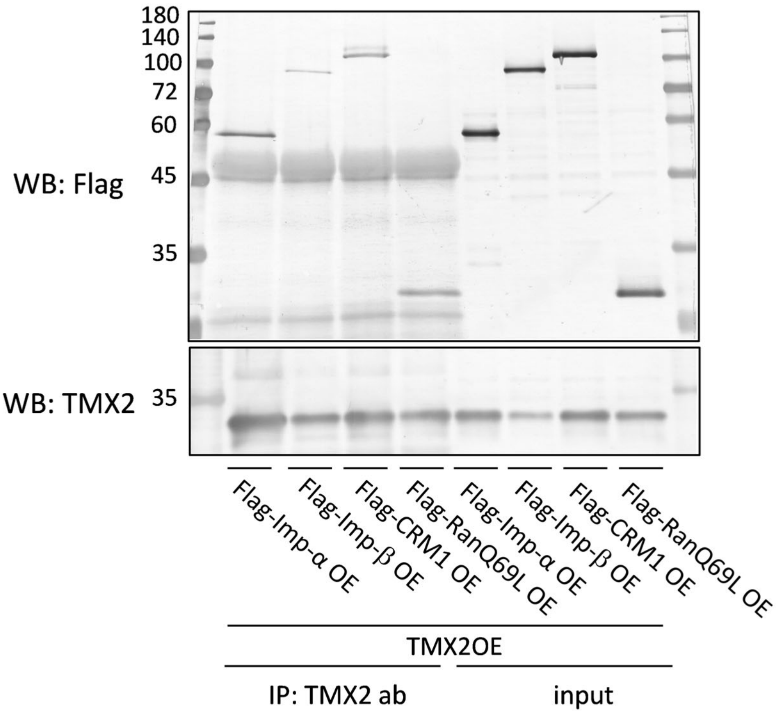

Supplementary Figure S4

The binding strength of overexpressed TMX2 with importin-α or CRM1 was comparable to that with importin-β or Ran.

Flag-importin-α, -importin-β, -CRM1, or -RanQ69L was co-overexpressed with TMX2 in HEK293 cells, and cell lysates were immunoprecipitated with anti-TMX2 antibody. The precipitates were detected with anti-Flag antibody.

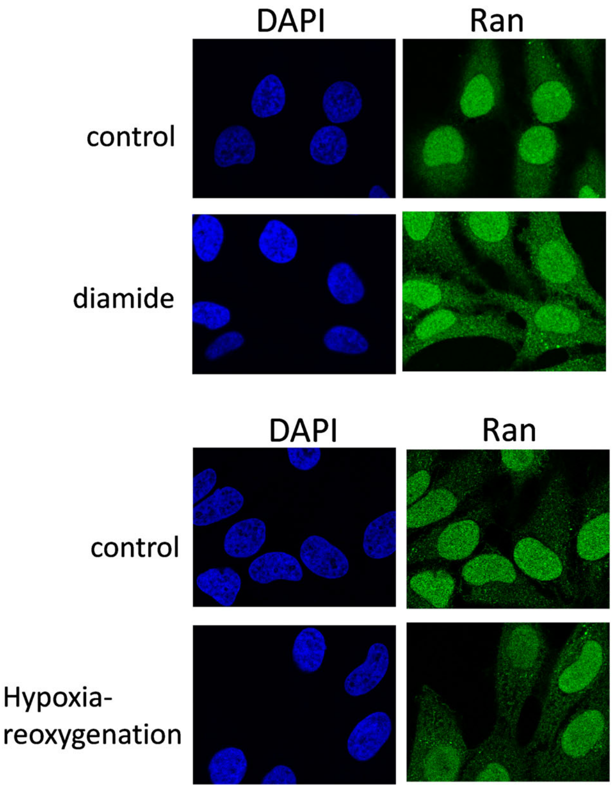

Supplementary Figure S5

Diamide, or hypoxia-reoxygenation decreased nuclear Ran levels.

Hela cells were treated with 200 μM diamide for 1 h, or cultured under 1% hypoxia for 4 h followed by reoxygenation with 21% oxygen for 20 min, and localization of Ran was observed by immunofluorescence microscopy.
